# Supplementary material for: Molecular Typing of Neisseria gonorrhoeae Clinical Isolates in Russia, 2018–2019: A Link Between penA Alleles and NG-MAST Types
Source: Pathogens. 2020 Nov 12;9(11):941. doi: 10.3390/pathogens9110941 (PMC7696878; doi:10.3390/pathogens9110941)
Supplement: Supplementary file 1 [file pathogens-09-00941-s001.pdf]

Table S1. Characteristics *of N. gonorrhoeae* isolates collected in the Russian Federation in 2018-2019

№№	Sample code	Region of isolation	Group	penA allele	ST (NG-MAST)	*porB*	*tbpB*	MIC (mg/L) PEN	MIC (mg/L) CRO	MIC (mg/L) PEN (group median)	MIC (mg/L) CRO (group median)	Genetic resistance determinants *ponA*	*mtrR* -35	*mtrR* -10	*porB*	*bla* TEM
1	14/19/09	Astrakhan	A	XV	1580	948	291	0.015	0.002			wt	wt	wt	wt	-
2	14/19/10	Astrakhan	A	XV	1580	948	291	0.015	0.002			wt	wt	wt	wt	-
3	14/18/11	Astrakhan	A	XV	1580	948	291	0.015	0.002			wt	wt	wt	wt	-
4	14/19/07	Astrakhan	A	XV	5714	738	6	0.06	0.002			wt	wt	wt	wt	-
5	20/19/05	Kaluga	A	XV	5714	738	6	0.12	0.002			wt	wt	wt	wt	-
6	20/19/06	Kaluga	A	XV	5714	738	6	0.06	0.002			wt	wt	wt	wt	-
7	14/18/16	Kaluga	A	XV	5714	738	6	0.03	0.002			wt	wt	wt	wt	-
8	07/19/01	Arkhangelsk	A	XV	9486	383	1714	0.03	0.002			wt	wt	wt	wt	-
9	07/19/03	Arkhangelsk	A	XV	9486	383	1714	0.06	0.002			wt	wt	wt	wt	-
10	07/19/04	Arkhangelsk	A	XV	9486	383	1714	0.06	0.002			wt	wt	wt	wt	-
11	07/19/08	Arkhangelsk	A	XV	9486	383	1714	0.25	0.002			wt	wt	wt	wt	-
12	07/19/09	Arkhangelsk	A	XV	9486	383	1714	0.25	0.002			wt	wt	wt	wt	-
13	07/19/13	Arkhangelsk	A	XV	9486	383	1714	0.06	0.002			wt	wt	wt	wt	-
14	07/19/23	Arkhangelsk	A	XV	9486	383	1714	0.12	0.002			wt	wt	wt	wt	-
15	07/18/04	Arkhangelsk	A	XV	9486	383	1714	0.015	0.002			wt	wt	wt	wt	-
16	07/18/05	Arkhangelsk	A	XV	9486	383	1714	0.015	0.002			wt	wt	wt	wt	-
17	07/18/23	Arkhangelsk	A	XV	9486	383	1714	0.015	0.002			wt	wt	wt	wt	-
18	07/18/25	Arkhangelsk	A	XV	9486	383	1714	0.015	0.002			wt	wt	wt	wt	-
19	07/18/29	Arkhangelsk	A	XV	9486	383	1714	0.015	0.002			wt	wt	wt	wt	-
20	07/18/30	Arkhangelsk	A	XV	9486	383	1714	0.015	0.002			wt	wt	wt	wt	-
21	12/19/08	Chuvash Republic	A	XV	14627	8482	6	0.015	0.002			wt	wt	wt	wt	-
22	12/18/14	Chuvash Republic	A	XV	14627	8482	6	0.03	0.002			wt	wt	wt	wt	-
23	12/18/15	Chuvash Republic	A	XV	14627	8482	6	0.03	0.002			wt	wt	wt	wt	-
24	07/19/10	Arkhangelsk	A	XV	14940	8661	1052	0.03	0.002			wt	wt	wt	wt	-
25	07/19/21	Arkhangelsk	A	XV	14940	8661	1052	0.03	0.002			wt	wt	wt	wt	-
26	20/19/13	Kaluga	A	XV	16174	9407	6	0.06	0.002			wt	wt	wt	wt	-
27	20/19/17	Kaluga	A	XV	16174	9407	6	0.015	0.002			wt	wt	wt	wt	-
28	07/18/26	Arkhangelsk	A	XV	16411	9533	6	0.015	0.002	0.03	0.002	wt	wt	wt	wt	-
29	07/18/27	Arkhangelsk	A	XV	16411	9533	6	0.015	0.002			wt	wt	wt	wt	-
30	12/19/06	Chuvash Republic	A	XV	18947	11053	6	0.003	0.002			wt	wt	wt	wt	-
31	12/19/11	Chuvash Republic	A	XV	18947	11053	6	0.03	0.002			wt	wt	wt	wt	-
32	12/19/23	Chuvash Republic	A	XV	18947	11053	6	0.03	0.002			wt	wt	wt	wt	-
33	09/19/08	Kazan	A	XV	19578	11415	175	0.015	0.002			wt	wt	wt	wt	-
34	09/19/09	Kazan	A	XV	19578	11415	175	0.06	0.002			wt	wt	wt	wt	-
35	09/19/10	Kazan	A	XV	19578	11415	175	0.015	0.002			wt	wt	wt	wt	-
36	09/19/12	Kazan	A	XV	19578	11415	175	0.015	0.002			wt	wt	wt	wt	-
37	19/18/16	Omsk	A	XV	5734	1614	118	0.015	0.002			wt	wt	wt	wt	-
38	19/18/21	Omsk	A	XV	5734	1614	118	0.015	0.002			wt	wt	wt	wt	-
39	14/18/22	Astrakhan	A	XV	17532	738	2599	1	0.004			wt	wt	wt	wt	-
40	20/19/11	Kaluga	A	XV	19648	11567	118	0.06	0.008			wt	wt	wt	wt	-
41	07/19/22	Arkhangelsk	A	XV	1993	1257	6	0.12	0.002			wt	wt	wt	wt	-
42	07/19/27	Arkhangelsk	A	XV	1993	1257	6	0.06	0.002			wt	wt	wt	wt	-
43	07/19/29	Arkhangelsk	A	XV	1993	1257	6	0.06	0.002			wt	wt	wt	wt	-
44	12/19/31	Chuvash Republic	A	XV	1993	1257	6	0.015	0.002			wt	wt	wt	wt	-
45	14/18/09	Astrakhan	A	XV	1993	1257	6	0.015	0.002			wt	wt	wt	wt	-
46	14/18/02	Astrakhan	A	XV	1993	1257	6	0.06	0.008			wt	wt	wt	wt	-
47	19/18/13	Omsk	A	XV	1993	1257	6	0.03	0.002			wt	wt	wt	wt	-
48	12/18/07	Chuvash Republic	A	XV	1993	1257	6	0.03	0.002			wt	wt	wt	wt	-
49	12/18/10	Chuvash Republic	A	XV	1993	1257	6	0.03	0.002			wt	wt	wt	wt	-
50	12/18/17	Chuvash Republic	A	XV	1993	1257	6	0.015	0.002			wt	wt	wt	wt	-
51	12/18/18	Chuvash Republic	A	XV	1993	1257	6	0.015	0.002			wt	wt	wt	wt	-
52	07/19/17	Arkhangelsk	A	XV	5042	1642	1052	0.015	0.004			wt	wt	wt	wt	-
53	07/18/17	Arkhangelsk	A	XV	5042	1642	1052	0.015	0.002			wt	wt	wt	wt	-
54	07/18/18	Arkhangelsk	A	XV	5042	1642	1052	0.015	0.002			wt	wt	wt	wt	-
55	07/18/19	Arkhangelsk	A	XV	5042	1642	1052	0.06	0.002			wt	wt	wt	wt	-
56	07/18/08	Arkhangelsk	B	I	228	23	27	0.06	0.004			wt	wt	wt	wt	-
57	02/18/01	Stavropol	B	I	228	23	27	0.06	0.004			wt	wt	wt	wt	-
58	02/18/02	Stavropol	B	I	228	23	27	0.06	0.004			wt	wt	wt	wt	-
59	02/18/03	Stavropol	B	I	228	23	27	0.03	0.004			wt	wt	wt	wt	-
60	02/18/04	Stavropol	B	I	228	23	27	0.03	0.004			wt	wt	wt	wt	-
61	02/18/05	Stavropol	B	I	228	23	27	0.06	0.004			wt	wt	wt	wt	-
62	02/18/06	Stavropol	B	I	228	23	27	0.06	0.004			wt	wt	wt	wt	-
63	02/18/07	Stavropol	B	I	228	23	27	0.06	0.004			wt	wt	wt	wt	-
64	02/18/08	Stavropol	B	I	228	23	27	0.06	0.004			wt	wt	wt	wt	-
65	02/18/09	Stavropol	B	I	228	23	27	0.06	0.004			wt	wt	wt	wt	-
66	02/18/10	Stavropol	B	I	228	23	27	0.03	0.004			wt	wt	wt	wt	-
67	02/18/13	Stavropol	B	I	228	23	27	0.03	0.004			wt	wt	wt	wt	-
68	02/18/14	Stavropol	B	I	228	23	27	0.06	0.004			wt	wt	wt	wt	-
69	02/18/16	Stavropol	B	I	228	23	27	0.06	0.004			wt	wt	wt	wt	-
70	07/18/11	Arkhangelsk	B	I	228	23	27	0.03	0.008			wt	wt	wt	wt	-
71	02/18/15	Stavropol	B	I	228	23	27	0.06	0.008			wt	wt	wt	wt	-
72	14/19/05	Astrakhan	B	I	228	23	27	0.5	0.004			wt	wt	wt	wt	-
73	07/19/18	Arkhangelsk	B	I	228	23	27	0.12	0.004			wt	wt	wt	wt	-
74	19/19/17	Omsk	B	I	228	23	27	0.25	0.004			wt	wt	wt	wt	-
75	12/19/24	Chuvash Republic	B	I	228	23	27	0.06	0.004			wt	wt	wt	wt	-
76	07/18/01	Arkhangelsk	B	I	807	37	27	0.06	0.004			wt	wt	wt	wt	-
77	07/18/03	Arkhangelsk	B	I	807	37	27	0.03	0.004			wt	wt	wt	wt	-
78	07/18/06	Arkhangelsk	B	I	807	37	27	0.06	0.004			wt	wt	wt	wt	-
79	12/18/16	Chuvash Republic	B	I	807	37	27	0.25	0.004			wt	wt	wt	wt	-
80	19/18/05	Omsk	B	I	807	37	27	0.25	0.008			wt	wt	wt	wt	-
81	12/18/09	Chuvash Republic	B	I	807	37	27	0.12	0.008			wt	wt	wt	wt	-
82	19/19/20	Omsk	B	I	807	37	27	0.03	0.004			wt	wt	wt	wt	-
83	12/19/02	Chuvash Republic	B	I	807	37	27	0.25	0.004			wt	wt	wt	wt	-
84	07/19/14	Omsk	B	I	807	37	27	0.5	0.004			wt	wt	wt	wt	-
85	07/19/16	Omsk	B	I	807	37	27	0.25	0.004	0.12	0.004	wt	wt	wt	wt	-
86	12/19/03	Chuvash Republic	B	I	807	37	27	0.25	0.008			wt	wt	wt	wt	-
87	12/19/10	Chuvash Republic	B	I	807	37	27	0.25	0.008			wt	wt	wt	wt	-
88	19/19/05	Omsk	B	I	807	37	27	0.5	0.015			wt	wt	wt	wt	-
89	19/19/09	Omsk	B	I	807	37	27	0.5	0.015			wt	wt	wt	wt	-
90	19/19/02	Omsk	B	I	807	37	27	1	0.03			wt	wt	wt	wt	-
91	19/19/01	Omsk	B	I	807	37	27	0.5	0.03			wt	wt	wt	wt	-
92	12/19/05	Chuvash Republic	B	I	5941	3547	27	0.25	0.004			wt	wt	wt	wt	-
93	12/19/07	Chuvash Republic	B	I	5941	3547	27	0.5	0.004			wt	wt	wt	wt	-
94	12/19/01	Chuvash Republic	B	I	5941	3547	27	0.25	0.008			wt	wt	wt	wt	-
95	14/18/01	Astrakhan	B	I	9570	5675	27	0.12	0.008			wt	wt	wt	wt	-
96	14/18/04	Astrakhan	B	I	9570	5675	27	0.12	0.008			wt	wt	wt	wt	-
97	14/18/06	Astrakhan	B	I	9570	5675	27	0.25	0.008			wt	wt	wt	wt	-
98	14/19/03	Astrakhan	B	I	9570	5675	27	0.5	0.004			wt	wt	wt	wt	-
99	12/19/04	Chuvash Republic	B	I	9570	5675	27	0.5	0.008			wt	wt	wt	wt	-
100	20/18/04	Kaluga	B	I	9576	5680	27	0.12	0.008			wt	wt	wt	wt	-
101	20/18/16	Kaluga	B	I	9576	5680	27	0.25	0.008			wt	wt	wt	wt	-
102	20/18/19	Kaluga	B	I	9576	5680	27	0.25	0.008			wt	wt	wt	wt	-
103	20/18/18	Kaluga	B	I	18246	5680	2798	0.25	0.008			wt	wt	wt	wt	-
104	20/18/20	Kaluga	B	I	18246	5680	2798	0.5	0.008			wt	wt	wt	wt	-
105	20/19/15	Kaluga	B	I	18894	11027	27	0.25	0.004			wt	wt	wt	wt	-
106	20/19/12	Kaluga	B	I	18894	11027	27	0.5	0.008			wt	wt	wt	wt	-
107	20/19/03	Kaluga	B	I	18894	11027	27	0.5	0.015			wt	wt	wt	wt	-
108	20/19/04	Kaluga	B	I	18894	11027	27	0.5	0.015			wt	wt	wt	wt	-
109	07/19/05	Arkhangelsk	B	I	18952	11057	27	2	0.015			wt	wt	wt	wt	-
110	12/19/09	Chuvash Republic	B	I	18953	11058	27	0.25	0.008			wt	wt	wt	wt	-
111	12/19/21	Chuvash Republic	B	I	18953	11058	27	0.12	0.008			wt	wt	wt	wt	-
112	12/19/26	Chuvash Republic	B	I	19572	5259	2961	0.12	0.004			wt	wt	wt	wt	-
113	12/19/16	Chuvash Republic	B	I	19572	5259	2961	0.03	0.008			wt	wt	wt	wt	-
114	12/19/28	Chuvash Republic	B	I	19572	5259	2961	0.06	0.008			wt	wt	wt	wt	-
115	20/19/01	Kaluga	C1	XXII	14020	421	2390	0.5	0.004			wt	wt	wt	wt	-
116	20/19/08	Kaluga	C1	XXII	14020	421	2390	0.5	0.004	0.375	0.004	wt	wt	wt	wt	-
117	19/19/12	Omsk	C1	XXII	17024	9921	189	0.12	0.004			wt	wt	wt	wt	-
118	14/19/08	Astrakhan	C1	XXII	19587	11424	674	0.25	0.004			wt	wt	wt	wt	-
119	12/19/30	Chuvash Republic	C2	XXII	19650	23	75	0.25	0.008	0.25	0.008	Leu421Pro	wt	wt	wt	-
120	12/19/32	Chuvash Republic	C2	XXII	19650	23	75	0.25	0.008			Leu421Pro	wt	wt	wt	-
121	12/18/03	Chuvash Republic	D	V	6226	1642	6	0.5	0.004			Leu421Pro	wt	wt	wt	-
122	12/18/01	Chuvash Republic	D	V	6226	1642	6	0.5	0.008			Leu421Pro	wt	wt	wt	-
123	12/18/02	Chuvash Republic	D	V	6226	1642	6	0.5	0.008			Leu421Pro	wt	wt	wt	-
124	12/18/06	Chuvash Republic	D	V	6226	1642	6	0.5	0.008			Leu421Pro	wt	wt	wt	-
125	12/18/19	Chuvash Republic	D	V	6226	1642	6	0.5	0.015			Leu421Pro	wt	wt	wt	-
126	12/19/13	Chuvash Republic	D	V	6226	1642	6	0.25	0.004			Leu421Pro	wt	wt	wt	-
127	12/19/12	Chuvash Republic	D	V	6226	1642	6	1	0.008			Leu421Pro	wt	wt	wt	-
128	12/19/17	Chuvash Republic	D	V	6226	1642	6	0.5	0.008			Leu421Pro	wt	wt	wt	-
129	12/19/18	Chuvash Republic	D	V	6238	23	6	0.5	0.008			Leu421Pro	wt	wt	wt	-
130	07/18/35	Arkhangelsk	D	V	14019	8094	4	0.12	0.02			Leu421Pro	wt	wt	wt	-
131	07/18/02	Arkhangelsk	D	V	14942	3450	4	0.25	0.008	0.5	0.008	Leu421Pro	wt	wt	wt	-
132	07/18/15	Arkhangelsk	D	V	14942	3450	4	0.12	0.008			Leu421Pro	wt	wt	wt	-
133	07/18/24	Arkhangelsk	D	V	14942	3450	4	0.12	0.008			Leu421Pro	wt	wt	wt	-
134	07/18/14	Arkhangelsk	D	V	14942	3450	4	0.12	0.015			Leu421Pro	wt	wt	wt	-
135	07/18/16	Arkhangelsk	D	V	14942	3450	4	0.25	0.015			Leu421Pro	wt	wt	wt	-
136	12/18/04	Chuvash Republic	D	V	17026	9922	6	0.5	0.008			Leu421Pro	wt	wt	wt	-
137	12/18/11	Chuvash Republic	D	V	17027	9923	6	0.5	0.008			Leu421Pro	wt	wt	wt	-
138	12/18/12	Chuvash Republic	D	V	17027	9923	6	1	0.008			Leu421Pro	wt	wt	wt	-
139	12/18/13	Chuvash Republic	D	V	17027	9923	6	0.5	0.008			Leu421Pro	wt	wt	wt	-
140	12/19/15	Chuvash Republic	D	V	17027	9923	6	0.12	0.008			Leu421Pro	wt	wt	wt	-
141	12/18/20	Chuvash Republic	D	V	17536	1228	958	0.5	0.004			Leu421Pro	wt	wt	wt	-
142	00/19/02	Moscow	E	XIII	16169	5136	18	2	0.008	2	0.012	Leu421Pro	wt	wt	wt	-
143	19/19/16	Omsk	E	XIII	16169	5136	18	2	0.015			Leu421Pro	wt	wt	wt	-
144	14/18/15	Astrakhan	F	II	16172	9404	629	0.25	0.004			Leu421Pro	wt	wt	wt	-
145	14/18/14	Astrakhan	F	II	16172	9404	629	0.25	0.008	0.25	0.004	Leu421Pro	wt	wt	wt	-
146	14/18/03	Astrakhan	F	II	17017	9919	273	0.25	0.004			Leu421Pro	wt	wt	wt	-
147	19/18/07	Omsk	G	IX	1451	931	6	1	0.015	1	0.023	Leu421Pro	wt	wt	Gly120Asp	-
148	19/18/04	Omsk	G	IX	1451	931	6	1	0.03			Leu421Pro	wt	wt	Gly120Asp	-
149	09/19/06	Omsk	H	V	18948	2020	32	1	0.008			Leu421Pro	wt	wt	Gly120Lys, Ala121Gly	-
150	09/19/01	Kazan	H	V	18948	2020	32	2	0.008			Leu421Pro	wt	wt	Gly120Lys, Ala121Gly	-
151	09/19/03	Kazan	H	V	18948	2020	32	4	0.015	1.5	0.015	Leu421Pro	wt	wt	Gly120Lys, Ala121Gly	-
152	09/19/04	Kazan	H	V	18948	2020	32	1	0.015			Leu421Pro	wt	wt	Gly120Lys, Ala121Gly	-
153	09/19/05	Kazan	H	V	18948	2020	32	4	0.015			Leu421Pro	wt	wt	Gly120Lys, Ala121Gly	-
154	09/19/02	Kazan	H	V	18948	2020	32	1	0.03			Leu421Pro	wt	wt	Gly120Lys, Ala121Gly	-
155	19/19/03	Omsk	I	V	13055	7289	6	1	0.004			Leu421Pro	delA	wt	wt	-
156	19/19/19	Omsk	I	V	13055	7289	6	1	0.004			Leu421Pro	delA	wt	wt	-
157	19/18/08	Omsk	I	V	13058	7589	6	2	0.015			Leu421Pro	delA	wt	wt	-
158	19/19/11	Omsk	I	V	13058	7589	6	1	0.008	1	0.008	Leu421Pro	delA	wt	wt	-
159	19/18/03	Omsk	I	V	16185	9411	6	1	0.015			Leu421Pro	delA	wt	wt	-
160	19/19/06	Omsk	I	V	18949	11054	27	2	0.015			Leu421Pro	delA	wt	wt	-
161	20/19/16	Kaluga	I	V	17534	10230	4	1	0.004			Leu421Pro	delA	wt	wt	-
162	19/18/09	Omsk	J	XXXIV (m)	3149	1903	110	2	0.06			Leu421Pro	delA	wt	Gly120Lys, Ala121Asn	-
163	07/18/33	Arkhangelsk	J	XXXIV (m)	5622	3411	110	0.25	0.12			Leu421Pro	delA	wt	Gly120Lys, Ala121Asn	-
164	14/18/12	Astrakhan	J	XXXIV (m)	10025	5904	110	0.5	0.015	1.5	0.045	Leu421Pro	delA	wt	Gly120Lys, Ala121Asn	-
165	14/18/05	Astrakhan	J	XXXIV (m)	10025	5904	110	1	0.03			Leu421Pro	delA	wt	Gly120Lys, Ala121Asn	-
166	14/19/01	Astrakhan	J	XXXIV (m)	10025	5904	110	2	0.03			Leu421Pro	delA	wt	Gly120Asp, Ala121Gly	-
167	14/19/02	Astrakhan	J	XXXIV (m)	10025	5904	110	2	0.12			Leu421Pro	delA	wt	Gly120Asp, Ala121Gly	-
168	20/18/01	Kaluga	K	44	12542	558	563	0.06	0.008			Leu421Pro	delA	wt	Gly120Asp	-
169	00/18/04	Moscow	K	44	12542	558	563	1	0.015	1	0.015	Leu421Pro	delA	wt	Gly120Asp	-
170	00/18/03	Moscow	K	44	12542	558	563	1	0.03			Leu421Pro	delA	wt	Gly120Asp	-
171	20/18/15	Kaluga	L	II	18245	10620	602	32	0.008	32	0.006	wt	wt	wt	wt	African type β-lactamase TEM-1
172	19/19/08	Omsk	L	XIV	18950	11055	27	32	0.004			wt	wt	wt	wt	African type β-lactamase TEM-1
173	00/19/01	Moscow		V	15906	6094	29	1	0.03			wt	wt	wt	wt	-
174	00/19/03	Moscow		II	6765	2078	29	2	0.008			wt	wt	wt	Gly120Lys, Ala121Asn	-
175	07/19/28	Arkhangelsk		II	16412	9534	189	2	0.008			wt	wt	wt	Gly120Asp	-
176	07/18/09	Arkhangelsk		IX	21	14	33	0.5	0.015			Leu421Pro	delA	wt	Gly120Lys, Ala121Asp	-
177	14/18/24	Astrakhan		XIII	17533	6094	1145	0.12	0.004			Leu421Pro	wt	wt	Ala121Ser	-
178	07/19/19	Arkhangelsk		I	13454	7783	812	0.5	0.004			Leu421Pro	delA	wt	wt	-
179	20/19/07	Kaluga		XVIII	19575	11027	75	0.5	0.008			Leu421Pro	delA	wt	wt	-
180	14/19/06	Astrakhan		II	19649	4367	72	1	0.008			Leu421Pro	delA	wt	wt	-
181	20/19/09	Kaluga		V	19574	11414	4	4	0.015			Leu421Pro	delA	wt	Gly120Lys, Ala121Asp	-
182	14/18/08	Astrakhan		44	17018	8484	563	0.25	0.008			Leu421Pro	delA	wt	wt	-
