# Supplementary material for: Molecular Typing of Neisseria gonorrhoeae Clinical Isolates in Russia, 2018–2019: A Link Between penA Alleles and NG-MAST Types
Source: Pathogens. 2020 Nov 12;9(11):941. doi: 10.3390/pathogens9110941 (PMC7696878; doi:10.3390/pathogens9110941)
Supplement: Supplementary file 2 [file pathogens-09-00941-s002.pdf]

Table S2. Results of linear regression analysis for distributing isolates obtained in Russia in 2018-2019 into groups (182 isolates)

Ceftriaxone group

Residuals:

Min	1Q	Median	3Q	Max
-1.6667	-0.5000	-0.1091	0.3839	2.2908

Coefficients:

Group	Coef.	Std. error	t value	*p* value
B	1.50698	0.12640	11.922	< 2e-16 ***
C1	0.89091	0.34923	2.551	0.011633 *
C2	1.89091	0.48545	3.895	0.000141 ***
D	1.94056	0.17299	11.218	< 2e-16 ***
E	2.34435	0.48545	4.829	3.07e-06 ***
F	1.22424	0.39983	3.062	0.002562 **
G	3.29780	0.48545	6.793	1.81e-10 ***
H	2.66217	0.28994	9.182	< 2e-16 ***
I	1.85101	0.27062	6.840	1.41e-10 ***
J	4.46447	0.28994	15.398	< 2e-16 ***
K	2.82884	0.39983	7.075	3.85e-11 ***
L	1.39091	0.48545	2.865	0.004700 **
Ungrouped	2.06298	0.23183	8.899	8.77e-16 ***
Intercept	-8.85669	0.09093	-97.398	< 2e-16 ***

*** $p < 0.001$, ** $p < 0.01$, * $p < 0.05$.
Residual standard error: 0.6744 on 168 degrees of freedom.
Multiple R-squared: 0.7254. Adjusted R-squared: 0.7041.
F-statistic: 34.13 on 13 and 168 DF, *p*-value: < 2.2e-16.

Penicillin group

Residuals:

Min	1Q	Median	3Q	Max
-3.3375	-1.0155	-0.0155	0.7757	5.0434

Coefficients:

Group	Coef.	Std. error	t value	*p* value
B	2.2676	0.2542	8.920	7.71e-16 ***
C1	3.2786	0.7024	4.668	6.20e-06 ***
C2	3.0434	0.9764	3.117	0.002150 **
D	3.5055	0.3479	10.075	< 2e-16 ***
E	6.0434	0.9764	6.189	4.47e-09 ***
F	3.0434	0.8042	3.784	0.000214 ***
G	5.0434	0.9764	5.165	6.74e-07 ***
H	5.8767	0.5832	10.077	< 2e-16 ***
I	5.3291	0.5443	9.790	< 2e-16 ***
J	5.0434	0.5832	8.648	4.03e-15 ***
K	3.6904	0.8042	4.589	8.68e-06 ***

L	10.0434	0.9764	10.286	< 2e-16 ***
Ungrouped	4.6375	0.4663	9.945	< 2e-16 ***
Intercept	-5.0434	0.1829	-27.575	< 2e-16 ***

*** $p < 0.001$, ** $p < 0.01$.
Residual standard error: 1.356 on 168 degrees of freedom.
Multiple R-squared: 0.7041. Adjusted R-squared: 0.6812
F-statistic: 30.75 on 13 and 168 DF, p-value: < 2.2e-16.
