# Supplementary material for: Molecular Typing of Neisseria gonorrhoeae Clinical Isolates in Russia, 2018–2019: A Link Between penA Alleles and NG-MAST Types
Source: Pathogens. 2020 Nov 12;9(11):941. doi: 10.3390/pathogens9110941 (PMC7696878; doi:10.3390/pathogens9110941)
Supplement: Supplementary file 3 [file pathogens-09-00941-s003.pdf]

Table S3. Results of regression analysis for MIC_{cro}.

Parameter	Estimate	Std. Error	t value	*p* value
penA I	-0.4920	0.4866	-1.011	0.3134
penA II	-0.5895	0.5001	-1.177	0.2409
penA IX	0.3925	0.5204	0.754	0.4517
penA V	0.0067	0.4873	0.014	0.9890
penA XIII	-0.1288	0.6162	-0.209	0.8347
penA XIV	-1.0978	0.8251	-1.331	0.1851
penA XV	-1.9887	0.4873	-4.081	6.9e-05***
penA XVIII	-0.0978	0.8251	-0.119	0.9058
penA XXII	-0.7645	0.5518	-1.385	0.1678
penA XXXIV	1.8412	0.5789	3.181	0.0017**
porB: Gly120Lys	0.6346	0.2563	2.476	0.0143*
porB: Gly120Asp	0.8075	0.4549	1.775	0.0777
Intercept	-6.8680	0.4788	-14.344	< 2e-16***

*** $p < 0.001$, ** $p < 0.01$, * $p < 0.05$.

Residual standard error: 0.6719 on 169 degrees of freedom.

Multiple R-squared: 0.7257. Adjusted R-squared: 0.7062.

F-statistic: 37.26 on 12 and 169 DF, *p*-value: < 2.2e-16
