# Supplementary material for: Molecular Typing of Neisseria gonorrhoeae Clinical Isolates in Russia, 2018–2019: A Link Between penA Alleles and NG-MAST Types
Source: Pathogens. 2020 Nov 12;9(11):941. doi: 10.3390/pathogens9110941 (PMC7696878; doi:10.3390/pathogens9110941)
Supplement: Supplementary file 4 [file pathogens-09-00941-s004.pdf]

Table S4. Results of regression analysis for MIC_{pen}.

Parameter	Estimate	Std. Error	t value	*p* value
penA I	2.2570	1.1394	1.981	0.0493*
penA II	3.2225	1.1325	2.845	0.0050**
penA IX	2.6265	1.0778	2.437	0.0159*
penA V	3.5508	1.0957	3.241	0.0014**
penA XIII	3.0208	1.3897	2.174	0.0311*
penA XIV	3.2225	2.2069	1.460	0.1461
penA XV	-0.0086	1.1448	-0.008	0.9940
penA XVIII	2.1375	1.6483	1.297	0.1965
penA XXII	3.1916	1.2557	2.542	0.0120*
penA XXXIV	0.3253	1.7735	0.183	0.8547
mtrR (-35)	1.8972	0.4730	4.011	9.11e-05***
bla_{TEM}	6.8122	1.4841	4.590	8.70e-06***
porB: Gly120Asp	2.1637	0.9184	2.356	0.0196*
porB: Ala121Gly	2.4914	0.5736	4.344	2.43e-05***
porB: Ala121Asn	2.8122	1.4841	1.895	0.0598
Intercept	-5.0347	1.1304	-4.454	1.54e-05***

*** $p < 0.001$, ** $p < 0.01$, * $p < 0.05$.

Residual standard error: 1.339 on 166 degrees of freedom.

Multiple R-squared: 0.7149. Adjusted R-squared: 0.6892.

F-statistic: 27.75 on 15 and 166 DF, *p*-value: < 2.2e-16.
